# Prediction accuracy of intraocular lens power calculation methods after laser refractive surgery

**DOI:** 10.1186/s12886-017-0439-x

**Published:** 2017-04-08

**Authors:** Yubo Wu, Songyu Liu, Rongfeng Liao

**Affiliations:** 1 Department of Ophthalmology, The First Affiliated Hospital of Anhui Medical University, No. 218 Jixi Road, Hefei, Anhui 230022 China; 2 Department of Ultrasound, The First Affiliated Hospital of Anhui Medical University, No. 218 Jixi Road, Hefei, Anhui 230022 China

**Keywords:** Biometry, Cataract surgery, Intraocular lens power calculation, Lasik

## Abstract

**Background:**

This study aimed to evaluate the prediction accuracy of postoperative refractions using partial coherence interferometry (IOL-Master) and applanation ultrasound (AL-3000) assisted with corneal topography (TMS-4) in eyes that had undergone myopic laser-assisted in situ keratomileusis (LASIK).

**Methods:**

Haigis-L formula, Koch–Maloney method using Haigis formula, Shammas clinically derived K-value (simulated keratometric value) correction (Shammas c.d.) using Haigis formula, and Shammas post-LASIK (Shammas-PL) formula were used in eyes with myopic LASIK. Constants were derived from the optimized constants in 133 virgin eyes. Refractive outcomes were determined by streak retinoscopy and subjective manifest refraction. Methods and formulas were evaluated by mean error (ME), standard deviation (SD), range of error, mean absolute error (MAE), median absolute error, 95% confidence interval of MAE, and percentage of eyes within ±0.5 diopter (D), ±1.0 D, and ±1.5 D of prediction.

**Results:**

SDs of the Haigis-L, Koch-Maloney method using the Haigis formula, Shammas c.d. using the Haigis formula, and the Shammas-PL formula using IOL-Master were 0.721, 0.695, 0.695, and 0.698; and those using AL-3000 assisted with TMS-4 were 0.782, 0.741, 0.743, and 0.778, respectively.

**Conclusions:**

No-history methods that corrected corneal power with measurements using IOL-Master were promising in myopic post-LASIK eyes, but still a gap in prediction accuracy exists between virgin eyes and post-LASIK eyes.

**Electronic supplementary material:**

The online version of this article (doi:10.1186/s12886-017-0439-x) contains supplementary material, which is available to authorized users.

## Background

Refractive outcomes after cataract surgeries and intraocular lens (IOL) implantation have always been a hot topic since the first IOL was implanted in 1949 [1], especially with the increasingly higher expectations of patients in recent years. In spite of its higher resolution, reproducibility, and repeatability of optical biometers [2], an ultrasonic device is irreplaceable in cases with severe opacities (e.g., corneal scar, mature and posterior subcapsular cataract, and vitreous hemorrhage) along the visual axis or fixation instability in most hospitals [3]. It is slightly doubtful that immersion ultrasound is more suitable than contact applanation ultrasound [4]. Ironically, the latter one is more widely used in China and many other countries, leading to increased attention paid to its prediction accuracy in IOL power calculation.

Patients who have undergone myopic laser-assisted in situ keratomileusis (LASIK) tend to have higher expectations regarding the refractive outcome. Intraocular lens calculation for them is much harder mainly because of the following reasons. First, it is hard for most devices to calculate the true post-LASIK corneal power using the corneal radius of curvature. This is due to the change in the relationship between the anterior and posterior curvatures of the cornea, making the standardized keratometric index inappropriate [5]. Moreover, when estimating the effective lens position (ELP), it is not wise to use the post-LASIK K-value (simulated keratometric value), as most of the third-generation formulas do [6].

Dozens of methods have been proposed to solve the problem. However, they can be hardly comparable with the calculation in virgin eyes. Most methods can be classified into two groups: methods that need data before the LASIK that patients underwent and methods that were based only on current measurements. Usually, the data before LASIK are not available or reliable, and literature has shown that no-history approaches are superior [7–10], which are widely used in clinical practice now. A series of methods that are used for calculating corrected corneal power (K_c_) are accepted for their accessibility and convenience. The present study concentrated on the three of them with different formulas: Haigis-L [7] formula; Koch–Maloney method [8], in which K_c_ = 1.114 × measured K – 6.1, using Haigis formula; Shammas c.d. [11], in which K_c_ = 1.14 × measured K – 6.8, using Haigis formula; and Shammas-PL formula [9]. Haigis-L is a formula that has been widely used and shown promising results. Shammas c.d. is normally used with the Shammas-PL formula. However, the formula is not routinely preinstalled in an optical device or ultrasonic device, making it a little inconvenient to use. Therefore, the question arose whether the Shammas-PL formula and Haigis formula, which predicted ELP from anterior chamber depth (ACD) and axial length (AL), were comparable.

This study prospectively evaluated three biometers (IOL-Master, AL-3000, and TMS-4) with four methods (Haigis-L formula, Koch-Maloney method using Haigis formula, Shammas c.d. using Haigis formula, and Shammas-PL formula) in post-LASIK eyes after optimization of constants in 133 virgin eyes.

## Methods

### Inclusion and exclusion criteria

This study recorded data from consecutive Chinese patients with virgin eyes who were hospitalized in the Department of Ophthalmology, The First Affiliated Hospital of Anhui Medical University between January 15, 2016, and May 1, 2016, and post-LASIK patients between July 15, 2015, and May 31, 2016. Patients who had undergone uneventful phacoemulsification cataract surgery by an experienced surgeon (Rongfeng Liao) with a temporal clear corneal incision, 3.0 mm in width, were included in the study. Eyes were included if they received in-the-bag placement of a monofocal IOL (920H, Rayner, Inc., London, UK; Adapt-AO, Bausch & Lomb, Inc., NY, USA; SN60WF, Alcon Laboratories, Inc., Hünenberg, Switzerland; or ZCB00, AMO, Inc., CA, USA). Eyes were excluded if any parameter relative to IOL power calculation (AL, K-value, and ACD) using IOL-Master, AL-3000, or TMS-4 could not be measured reliably. Other exclusion criteria included corneal astigmatism more than 1.5 diopter (D), postoperative best-corrected visual acuity (BCVA) less than 20/40, previous ocular surgeries except myopic LASIK, combined surgery, intraoperative and postoperative complications, active ocular infection, and systemic diseases that might have affected eyes. Patients who had a follow-up time less than 3 months were also excluded. If a patient had both eyes operated, only the first qualified eye was included in the study. This paper was approved by the medical ethics committee of the hospital. The study adhered to the Declaration of Helsinki, and informed consent was obtained from all patients.

### Preoperative and postoperative assessment

Preoperative assessment included examinations from IOL-Master (version 5.5, Carl Zeiss Meditec, Inc., Jena, Germany, keratometric index: 1.3375), TMS-4 (Topographic Modeling System, Tomey, Inc., Nagoya, Japan, keratometric index: 1.3375), and AL-3000 (Bio & Pachy Meter AL-3000, Tomey, Inc., Nagoya, Japan). The examination of AL-3000 was performed last to avoid corneal indentation and maintain the integrity of the corneal epithelium when other preoperative measurements were gauged. The sequence between IOL-Master and TMS-4 was random. Patients were asked to blink just before measurements were taken. Pupil sizes during preoperative assessments were normal [12]. All preoperative examinations were carried out by the first author, who was trained and qualified according to the manufacturer’s recommendation. The follow-up points were 1 week, 1 month, and 3 months after the surgery when postoperative assessments using slit-lamp biomicroscopy, auto refractometer, streak retinoscopy, and subjective manifest refraction were implemented. The data in this article were from the 3-month point.

### Optimization of constants and prediction error

In virgin eyes, the constants were optimized for each IOL model, formula, and biometer to make the mean error equal to zero. Formulas were programmed in the Excel software (version 12.0, 2007, Microsoft Corp., WA, USA) by the first author and carefully checked against IOL-Master. Hoffer Q [13], Holladay I [14], and SRK/T [15] were optimized by the function of What If Analysis in the Excel software, and a double linear regression analysis was used for Haigis [16]. However, Constants of SRK II [17] were not optimized because it was considered to be an outdated regression formula [18]. Then, the optimized constants were used in post-LASIK eyes. As for C constant in the Shammas-PL formula, the conversion eq. (C = 0.5835 × A **–** 64.40) was used to calculate it from the optimized A constant of SRK/T [9]. The prediction error was defined as the stable postoperative manifest refraction at BCVA in spherical equivalent (SE) minus predicted SE, which means that a positive prediction error indicated a hyperopic shift. Meanwhile, the absolute error was defined as the absolute value of the prediction error.

### Statistical analysis

SPSS (version 22.00, SPSS, Inc., IL, USA) was used for statistical analysis. Standard deviations (SDs) were calculated, and F tests were used for determining the significant differences between formulas and methods. *P* values less than 0.05 were considered to be statistically significant.

## Results

Preoperative data of 176 virgin eyes of 176 patients and 10 post-LASIK eyes of 10 post-LASIK patients were collected. Finally, 133 virgin eyes of 133 patients and 10 post-LASIK eyes of 10 post-LASIK patients were analyzed in this study as a result of losing to follow-up (28 eyes), complications (4 eyes), or postoperative BCVA less than 20/40 (11 eyes).

The demographics, mean values (± SDs), and ranges measured by IOL-Master were as follows: virgin eyes—62 males (46.6%), 71 females (53.4%), 63 oculi dexter (47.4%), 70 oculi sinister (52.6%), 36 eyes (27.1%) implanted 920H IOL, 33 eyes (24.8%) implanted Adapt-AO IOL, 33 eyes (24.8%) implanted SN60WF IOL, 31 eyes (23.3%) implanted ZCB00 IOL, age 68.2 ± 10.3 (range 41.0–89.0), AL 24.42 ± 2.40 mm (range 21.11–33.58 mm), average K 43.93 ± 1.72 D (range 39.88–47.68 D), ACD 3.16 ± 0.44 mm (range 1.97–4.05 mm), and IOL power 18.66 ± 5.81 D (range − 5–27 D); post-LASIK eyes—2 males (20.0%), 8 females (80.0%), 5 oculi dexter (50.0%), 5 oculi sinister (50.0%), 4 eyes (40.0%) implanted 920H IOL, 2 eyes (20.0%) implanted Adapt-AO IOL, 1 eye (10.0%) implanted SN60WF IOL, 3 eyes (30.0%) implanted ZCB00 IOL, age 50.3 ± 9.0 (range 40.0–69.0), AL 30.06 ± 2.87 mm (range 25.46–34.08 mm), average K 36.35 ± 0.77 D (range 35.09–37.84 D), ACD 3.34 ± 0.41 mm (range 2.75–3.86 mm), and IOL power 15.70 ± 6.24 D (range 8–26 D).

**Fig. 1:**
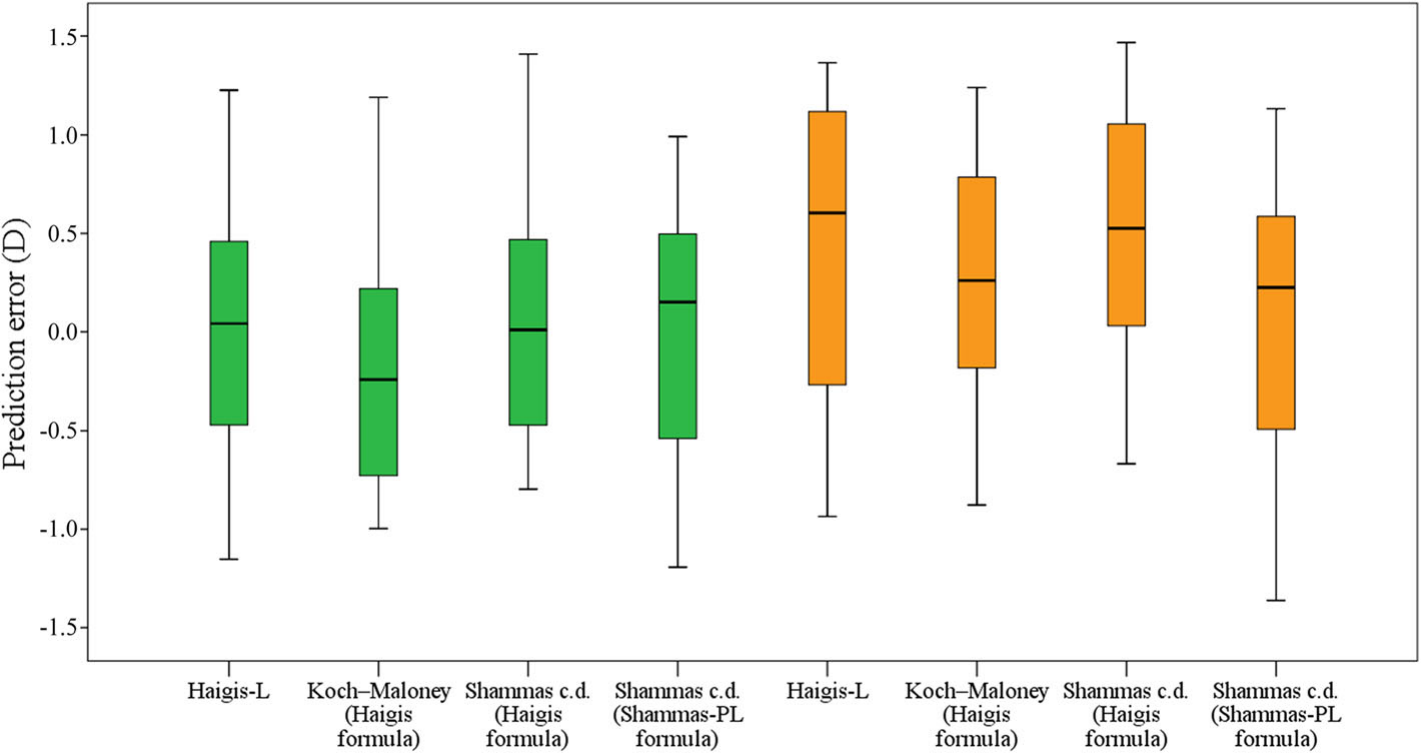
Box plot of prediction errors for each method. *Green color* represents measurements from IOL-Master. *Yellow color* represents measurements from AL-3000 and TMS-4

Table 1 shows the results for post-LASIK eyes measured by IOL-Master and AL-3000 assisted with TMS-4, and Fig. 1 shows the box plot of the prediction errors. Table 2 shows the optimized IOL constants used in this study.

**Table 1:** Results of 10 post-LASIK eyes

Method	ME	SD	Range	MAE	MedAE	95% CI	± 0.5 D	± 1.0 D	± 1.5 D
IOL-Master									
Haigis-L	0.01	0.721^a^	– 1.15–1.23	0.604	0.465	0.361–0.847	6	8	10
Koch–Maloney (Haigis formula)	– 0.15	0.695	– 1.00–1.19	0.557	0.510	0.312–0.842	5	9	10
Shammas c.d. (Haigis formula)	0.09	0.695	– 0.80–1.41	0.557	0.470	0.283–0.831	6	9	10
Shammas c.d. (Shammas-PL formula)	– 0.02	0.698^a^	– 1.19–0.99	0.558	0.519	0.334–0.822	5	9	10
AL-3000 assisted with TMS-4									
Haigis-L	0.45	0.782	– 0.94–1.37	0.774	0.876	0.480–1.067	4	7	10
Koch–Maloney (Haigis formula)	0.25	0.741	– 0.88–1.24	0.627	0.781	0.324–0.931	4	8	10
Shammas c.d. (Haigis formula)	0.49	0.743	– 0.67–1.47	0.734	0.708	0.396–1.072	3	6	10
Shammas c.d. (Shammas-PL formula)	0.03	0.778	– 1.36–1.13	0.653	0.583	0.392–0.914	4	8	10

*ME* mean error, *SD* standard deviation, *MAE* mean absolute error, *MedAE* median absolute error, *95% CI*, 95% confidence interval of mean absolute error; ±0.5 D, ±1.0 D, and ±1.5 D = number of refractions within 0.5 D, 1.0 D, and 1.5 D of prediction

^a^Worse than Haigis with measurements using IOL-Master in all 133 virgin eyes (*P* < 0.05)

**Table 2:** Intraocular lens constants used for formulas

	Formula			
	Haigis			Shammas-PL
IOL model	a0	a1	a2	C
IOL-Master				
920H	0.154	0.166	0.166	4.612
Adapt-AO	−1.418	0.629	0.176	4.710
SN60WF	1.948	0.376	0.076	4.883
ZCB00	−3.031	0.331	0.309	5.403
AL-3000 assisted with TMS-4				
920H	−1.068	0.209	0.191	4.176
Adapt-AO	−0.319	0.563	0.106	4.114
SN60WF	3.091	0.319	0.009	4.432
ZCB00	0.632	0.614	0.095	4.931

In post-LASIK eyes, the refractive errors of most eyes were within 1.0 D, and all of them were within 1.5 D. The statistics also showed that the Haigis formula and Shammas-PL formula were interchangeable with Shammas clinically derived K-value correction. Moreover, when measurements were performed using IOL-Master, tiny mean errors (MEs) with little clinical significance were observed, with Koch-Maloney method using Haigis formula tended to result in a little myopic shift. However, applanation ultrasound did not result in ignorable hyperopic surprise except when Shammas c.d. and Shammas-PL formula were used. Significant differences of SDs were found between the results of Haigis-L and Shammas c.d. with the Shammas-PL formula and the results of Haigis in virgin eyes with measurements using IOL-Master. In the data of AL-3000 assisted with TMS-4, no significant difference was found between methods in post-LASIK eyes and Haigis in virgin eyes because of the larger SD of Haigis in virgin eyes.

## Discussion

Comparing methods with optimized constants in post-LASIK eyes is of great significance. It is a must to show their true prediction ability and avoid other interference factors. This novel study evaluated methods in post-LASIK eyes with optimized constants under certain circumstances. It is widely recognized that AL measured using applanation ultrasound is shorter than that measured using a partial coherence interferometry (PCI) device [19]. Applanation ultrasound achieved fewer number of eyes within 0.5 D and 1.0 D, although the differences of SDs were not so obvious. Hyperopic surprises in eyes after myopic LASIK have been a concern for many years [11], but now, it is possible to solve this problem using modern methods and PCI devices and even without preoperative data, albeit with larger SDs. A possible explanation for this is that all these corneal power correction approaches assume a one-to-one relation between the measured K-value and corrected K-value and do not take the extent of ablation into consideration. Literature shows that the error is directly proportional to the extent of keratectomy [20]. However, when applanation ultrasound was used, no significant difference was found between virgin eyes and post-LASIK eyes because of the larger SD of Haigis in virgin eyes. It might be due to the poorer reproducibility and repeatability of applanation ultrasound while measuring AL, and weaker correlation when constants of Haigis were optimized. Hyperopic shift was most likely to be minimized with Shammas c.d. and Shammas-PL formula. As they were observed in other three methods, it might be more suitable to combine the Haigis formula with a PCI device than applanation ultrasound in post-LASIK eyes. Nevertheless, as all refractive errors are within 1.5 D, most post-LASIK patients are likely to be acceptable with refractive outcomes.

More importantly, the errorless measurement of corneal power is a premise. The present study found that it was likely to get K-values with great variations when measurements were repeated several times in post-LASIK eyes with decentered ablation. The possible reason is that, sometimes, some of the measurement points are within the ablation area, and sometimes not. In these cases, asking patients to focus stably is important, and corneal topography may help a lot. For them, corneal topography can directly show the ablation area and its relationship with the corneal vertex and the pupil. If the ablation area still includes most area of the pupil, and patients can recall that they were relatively satisfied with the LASIK just after the operation, it indicates that their optical axes are still inside the ablation area. Thus, patients can be asked to focus on another point or reduce the radius of measurement, if necessary, to ensure that all measurement points are within the ablation area and achieve the most accurate measurement.

The results of the present study were comparable to those of many previous studies. Haigis [7] found that the ME of the Haigis-L formula was not significantly different from 0. Chen et al. [10] found through meta-analysis that Shammas c.d. with the Shammas-PL formula outperformed the Haigis-L formula in eyes after laser refractive surgery. Jin et al. [21] found that the Koch-Maloney method achieved lower MAE than Shammas c.d. with the Haigis formula, but the difference was not statistically significant. However, the constants in their study were not optimized for each IOL model. Jin et al. [22] found that when the Koch–Maloney method was used in Chinese eyes, an offset value of −6.2 was more appropriate than −6.1. However, in the present study, the results from IOL-Master did not show the same tendency. The possible reason was that the number of cases in this study and previous studies was relatively small, and the difference between two offsets was tiny.

This study had several limitations. First, four IOL models were used instead of one. The main reason was that if only one IOL model were included, it would leave too few post-LASIK eyes to be analyzed, and the influence on clinical decisions was not the intention of this study. Meanwhile, many scholars believe that it is acceptable provided that constants were optimized for each IOL model. Besides, the study group was relatively small.

Nowadays, more and more sophisticated devices that can gauge more parameters of the cornea, such as Pentacam, are continuously being invented. These devices make it possible to measure corneal power directly in post-LASIK eyes. Future studies will explore whether it is possible to achieve more desirable outcomes when the extent of ablation is taken into account and the corneal power is measured more directly using these devices. The prediction accuracy of post-LASIK eyes and virgin eyes may be the same with advanced algorithm and technology.

## Conclusions

In post-LASIK eyes, no-history methods that corrected the corneal power with measurements using IOL-Master are promising, but still a gap in prediction accuracy exists between virgin eyes and post-LASIK eyes. However, when the Haigis formula is used with measurements using applanation ultrasound and corneal topography, it tends to result in hyperopic shifts. The results suggest an acceptable refractive outcome can be achieved in most patients.

## Additional file

Additional file 1: Results of measurements and prediction errors for each case. This file shows all necessary data that was used for calculation in this article. (XLSX 11 kb)

## Abbreviations

ACD: Anterior chamber depth
AL: Axial length
BCVA: Best-corrected visual acuity
D: Diopter
ELP: Effective lens position
IOL: Intraocular lens
LASIK: laser-assisted in situ keratomileusis
MAE: Mean absolute error
ME: mean error
PCI: Partial coherence interferometry
SD: standard deviation
SE: Spherical equivalent
